# Oxaliplatin retains HMGB1 intranuclearly and ameliorates collagen type II-induced arthritis

**DOI:** 10.1186/ar2347

**Published:** 2008-01-07

**Authors:** Therese Östberg, Heidi Wähämaa, Karin Palmblad, Norimasa Ito, Pernilla Stridh, Maria Shoshan, Michael T Lotze, Helena Erlandsson Harris, Ulf Andersson

**Affiliations:** 1 Department of Woman and Child Health, Pediatric Rheumatology Research Unit, Karolinska Institutet/Karolinska University Hospital, 171 176 Stockholm, Sweden; 2 Department of Medicine, Rheumatology Unit, Karolinska Institutet/Karolinska University Hospital, 171 76 Stockholm, Sweden; 3 Surgery and Bioengineering, DAMP Laboratory, Hillman Cancer Center, Research Pavilion, University of Pittsburg, Pittsburg, PA 15213, USA; 4 Department of Clinical Neuroscience, Karolinska Institutet/Karolinska University Hospital, 171 76 Stockholm, Sweden; 5 Department of Oncology Pathology, Karolinska Institutet/Karolinska University Hospital, 171 76 Stockholm, Sweden

## Abstract

**Introduction:**

High mobility group box chromosomal protein 1 (HMGB1) is a nuclear protein that acts as a pro-inflammatory mediator following extracellular release. The protein is aberrantly expressed extracellularly in the settings of clinical and experimental synovitis. Therapy based on HMGB1 antagonists has shown encouraging results in experimental arthritis and warrants further scientific exploration using independent methods. In the present study we asked whether nuclear sequestration of HMGB1 preventing HMGB1 release would be beneficial for synovitis treatment.

**Methods:**

Oxaliplatin-based therapy was evaluated in collagen type II-induced arthritis in DBA/1 mice by clinical scoring and immunostaining of articular tissue. Oxaliplatin is an antineoplastic platinum-based compound that generates DNA adducts which tightly bind HMGB1. Secretion and intracellular location of HMGB1 were assessed by a novel HMGB1-specific ELISPOT assay and immunofluorescent staining.

**Results:**

Intraperitoneal injections of oxaliplatin in early collagen type II-induced arthritis trapped HMGB1 with a distinct biphasic response pattern. Oxaliplatin therapy showed beneficial results for approximately 1 week. Microscopic evaluation of synovitis during this period showed strong nuclear HMGB1 staining in the oxaliplatin treated animals with much lower quantities of extracellular HMGB1 when compared to control treated animals. Furthermore, cellular infiltration, as well as cartilage and bone damage, were all reduced in the oxaliplatin treated group. A dramatic and as yet unexplained clinical relapse occurred later in the oxaliplatin exposed animals, which coincided with a massive synovial tissue expression of extracellular HMGB1 in all treated animals. This rebound-like reaction was also accompanied by a significantly increased incidence of arthritis in the oxaliplatin treated group. These results indicate a distinct temporal and spatial relationship between the clinical course of disease and the cellular localization of HMGB1. Beneficial effects were noted when extracellular HMGB1 expression was low, while severe inflammation coincided with substantial extracellular synovial HMGB1 expression.

**Conclusion:**

Therapeutic compounds like oxaliplatin and gold salts share a capacity to inhibit nuclear HMGB1 release and to ameliorate the course of synovial inflammation. These observations support the hypothesis that HMGB1 plays an important functional role in the pathogenesis of arthritis and may represent a novel target molecule for therapy.

## Introduction

Major progress has been achieved during the last decade in the treatment of patients with several chronic inflammatory diseases using biological therapies targeting the cytokines tumor necrosis factor (TNF) or interleukin (IL)-1β. These advances warrant a search for additional endogenous target molecules in inflammatory cascades suitable for therapeutic intervention. The notion that high mobility group box 1 protein (HMGB1) may constitute one such candidate molecule forms the background for the present work.

HMGB1 is a nuclear, non-histone DNA-binding protein with extranuclear roles as well. HMGB1 mediates multiple functions depending on localization and molecular context (reviewed in [1,2]). The protein is remarkably preserved among species and is expressed in all nucleated cells, where it regulates structural and transcriptional activities [3,4]. HMGB1 may in addition be translocated/secreted to extracellular sites, where it unexpectedly acts a mediator of inflammation and tissue repair [2,5,6]. HMGB1 is either actively secreted or passively released from dying cells [5,7]. The extracellular transport of HMGB1 occurs by a non-conventional pathway that differs from that of most other secreted pro-inflammatory proteins [8-10]. Inside the cell, HMGB1 can shuttle between the nucleus and cytoplasm. With activation, however, acetylation and phosphorylation alter the charge of HMGB1 and its interaction with chromatin. These post-translational modifications cause HMGB1 relocation to the cytosol [8,11]. A specific ABC transporter, MRP1, then translocates HMGB1 into secretory lysosomes for extracellular exocytosis. The transport by MRP1 requires covalent linkage of HMGB1 to glutathione [12].

Extracellular HMGB1 may play a major role in the pathogenesis of synovitis, since it is a potent promotor of macrophage activation including induction of TNF as well as IL-1 synthesis and other pro-inflammatory mediators [13-15]; Extracellular HMGB1 is abundantly expressed in serum, in synovitis and in intra-articular fluid of patients with rheumatoid arthritis as well as in experimental arthritis [16-18].

HMGB1 localization in normal synovial tissue is almost exclusively restricted to the nuclear compartment. Cell membrane-expressed HMGB1 in tumors promotes local tissue invasion. It may also have a causal connection to pannus-induced structural damage, since HMGB1 is strongly displayed both at a protein and mRNA levels in this tissue in collagen-induced arthritis (CIA) [19]. Intra-articular rHMGB1 injections in mice cause prolonged, destructive arthritis [20]. HMGB1 has been targeted successfully in CIA using antagonists including anti-HMGB1 antibodies or truncated HMGB1 (A box peptide). Furthermore, treatment with sRAGE, the soluble form of the receptor for advanced glycated proteins (RAGE), which is a HMGB1-signaling receptor, has also been beneficial in CIA [21]. Therapy based on truncated thrombomodulin ameliorates several forms of experimental arthritides [22]. It has been demonstrated that thrombomodulin binds to HMGB1 and neutralizes its extracellular pro-inflammatory activity [23].

Preventing extracellular HMGB1 release by intracellular sequestration provides a novel strategy to evaluate a functional role of HMGB1 in the pathogenesis of synovitis. It is well established that binding of HMGB1 to undamaged DNA is a rapid and transient process. HMGB1 constantly shuttles within the nucleus and between the nuclear and cytoplasmic compartments [8]. In contrast, HMGB1 binds tightly to sites of distorted DNA [24]. Covalent DNA adducts generated by the platinating anti-tumor drugs cisplatin and oxaliplatin sequester nuclear HMGB1 [25,26]. In the present study, we used the platinum cytostatic compound oxaliplatin, which is a more recently developed analogue of cisplatin, to study aspects of HMGB1 biology in CIA in mice and in cell culture experiments.

## Materials and methods

### Induction of CIA

DBA/1 mice, 18–22 g were obtained from Harlan Netherlands B.V. (Horst, The Netherlands). Animals were housed in specific pathogen-free facilities at Karolinska University Hospital, Stockholm, Sweden. All experimental procedures were approved by the Stockholm North Ethical Committee, Sweden. The mice were housed five animals per cage, had free access to water and standard rodent chow. A 12-h light/dark cycle was maintained at all times.

Collagen type II was prepared from bovine nasal cartilage and collagen emulsion was prepared as previously described [27-29]. Arthritis was induced as previously described [29]. Mice were observed daily for erythema and swelling of the joints. The intraphalangeal joints of digits, metacarpophalangeal and wrist in the forepaw and ankle joint in hind paw were each considered as one category of joint. Individual paws were evaluated by a score ranging from 0–3: 0 = no signs of arthritis, 1 = one type of joint affected, 2 = two types of joints affected, and 3 = the entire paw affected. Thus the maximal score for each animal was 12. Evaluation of arthritis was performed by staff members blinded to the identity of the animals.

### Treatment of CIA with oxaliplatin

The first experiment was based on therapy with oxaliplatin (Eloxatin, Aventis Pharma, UK) diluted in sterile water (according to the manufacturer's instructions) to a concentration of 10 mg/kg administrated as a single injection given either intraperitoneally (IP) or intravenously (IV) on day 31 post-immunisation (PI). The animals were followed until day 40 PI. In the second treatment protocol, oxaliplatin (10 mg/kg) was administered IP on day 31 and 37 PI. Animals were followed until day 44 PI. The concentration of oxaliplatin in plasma has been reported to drop rapidly after distribution, reaching a peak concentration of 37 μM within 5 min after administration and declining to 5 μM after a few h. Oxaliplatin assessments after 24 h and 48 h demonstrated plasma levels of 2 μM and 1 μM, respectively [30]. Intravenous administration was not performed in the second experiment since IV and IP injections gave similar results in the initial experiment. Injections with NaCl served as control vehicle in both studies instead of sterile water to avoid hemolytic effects.

### Immunohistochemical analysis

Paws collected day 36 and 44 where fixed in Zamboni solution (paraformaldehyde and picric acid). Paws were dissected and decalcified as previously described [31]. Cryosections of 7 μm were collected on Superfrost slides (Menzel GmbH & Co KG, Braunschweig, Germany).

Sections were stained with hematoxylin and Safranin O (0.1%). Cell infiltration was graded on a scale ranging from 0 (no infiltration) to 3 (severe inflamed joint). Cartilage destruction was scored on a scale from 0 to 3 ranging from no abnormalities to completely destroyed or destained cartilage. Bone erosion was graded on a scale from 0 (normal bone appearance) to 3 (fully eroded cortical bone and ankylosis).

In addition, serial sections were stained for HMGB1 as previously described [19]. In short, slides were incubated over night with a primary peptide affinity-purified polyclonal rabbit anti-HMGB1 antibody (cat. no. 556528, Pharmingen, San Diego, CA, USA). Slides were thereafter incubated with biotin-labeled donkey anti-rabbit antibody (cat. no. 711-066-152, Jackson ImmunoResearch Lab, West Grove, PA, USA), Fab_2_-fragmented. The substrate diaminobenzidine (DAB; Peroxidase Substrate Kit, Vector Laboratories Inc.) was added to develop the slides. Sections were counterstained with Mayer's hematoxylin solution (Histolab, Gothenburg, Sweden). Semi-quantitative assessment of the frequency of positively stained cells in the synovial tissue was graded using the following scale: 0, no positively stained cells; 1, <0.5% stained cells; 2, 0.5–5% stained cells; 3, 5–20% stained cells; 4, 20–50% stained cells; 5, >50% stained cells.

### T-cell proliferation

T-cell proliferation was performed as previously described [32]. In short, inguinal lymph nodes (LN) cells from male mice immunized with ovalbumin (OVA, Sigma Aldrich, Steinheim, Germany) in CFA were cultured *in vitro *for 96 h at 1 × 10^6 ^cells/ml, in the presence of OVA (10 μg/ml or 50 μg/ml), phosphate-buffered saline (PBS) or ConA (2 μg/ml) (Sigma Aldrich) oxaliplatin (Sanofi-Synthlabo, Malvern, PA, USA) 0.6–5 μM. [^3^H]-thymidine (Perkin Elmer Life Sciences Inc., Boston, MA, USA) 1 μCi/well, was added for the final 16 h of culture. [^3^H]-thymidine incorporation was measured as counts per min (cpm) in a Wallac Trillux 1450 microbeta counter. Cell viability was assessed at initiation and harvest of cultures and determined to be 87–100% using Trypan blue (Merck, Darmstadt, Germany) exclusion and Cytotoxicity Detection Kit (LDH) (no. 11644793001, Roche, Mannheim, Germany) according to the manufacturer's instructions. There were no differences observed in viability between oxaliplatin treated cells and control cells.

### Cell culture

Murine macrophage-like RAW 264.7 cells were cultured in DMEM or RPMI medium (Gibco, Paisley, Scotland, UK) supplemented with 5–10% fetal calf serum (FCS), 100 U/ml Penicillin, 100 μg/ml Streptomycin, 2 mM L-glutamine and β-mercaptoethanol (Life Technologies, Paisley, Scotland). At a confluence of 80–90%, cells were harvested by flushing with medium.

### Immunofluorescence confocal microscopy

RAW 264.7 cells were plated in four-chambered cover slides (Lab-Tek, Nalge, Rochester, NY, USA), 5 × 10^4 ^cells/ml, incubated overnight. Cells were washed with PBS, new media and 0.5 μM oxaliplatin was added and stimulated with 10 μg/ml LPS L-6529 (Sigma Chemical Co., St Louis, MO, USA), 100 U/ml mouse rIFN-γ (Sigma) for 24 h. After incubation, cells were fixed with 2% paraformaldehyde. Cells were permeabilized in 0.1% Triton X-100 in PBS supplemented with 0.5% bovine serum albumen (BSA) and 0.15% glycine for 30 min. Cells were blocked in 20% normal goat serum in PBS containing 0.5% bovine serum albumin, 0.15% glycine for 40 min. Anti-HMGB1 antibody (generated by Sigma Antibody Service, St Louis, MO, USA; 1:500) was added to cells and incubated at room temperature for 1 h. Goat anti-rabbit Alexa 488 (1:500; Invitrogen, Carlsbad, CA, USA) and rhodamine–phalloidin (1:250; Invitrogen) was added for 1 h at room temperature. Cells were washed with 0.5% BSA and 0.15% glycine between all incubations steps, followed by a final wash in PBS. Nuclei were counterstained with Hoechst (0.01%). Cells were mounted using gelvatol (23 g poly(vinyl alcohol)-2000, 50 ml glycerol and 0.1% sodium azide to 100 ml PBS), then viewed with a confocal scanning fluorescence microscope (Olympus Fluoview, Malvern, NY, USA).

### Elispot

The Elispot assay was performed as previously described [33]. RAW 264.7 cells, oxaliplatin (Sanofi-Synthlabo) 0.03–0.5 μM, were added to the plate 24 h prior stimulation with 10 μg/ml LPS L-6529 (Sigma) and 10 ng/ml mouse rIFNγ (Sigma). The plates were analyzed in an AID EliSpot Reader System (AID, Strassberg, Germany). Cell viability was assessed at initiation and harvest of cultures, and determined to be 95–100% using Trypan blue (Merck) exclusion and MTT (3-(4,5-dimethylthiazol-2-yl)-2,5-diphenyl tetrazolium bromide)-based cell growth determination kit (no. CGD1-1KT; Sigma) according to the manufacturer's instructions.

### Statistical analysis

Data were analyzed for statistical significance using the Mann–Whitney U test for independent groups when comparing arthritis scores. Fisher's exact test was used to compare differences in arthritis incidence between the groups. A p value < 0.05 was considered statistically significant.

## Results

### Clinical effects on CIA by oxaliplatin treatment

**Figure 1:**
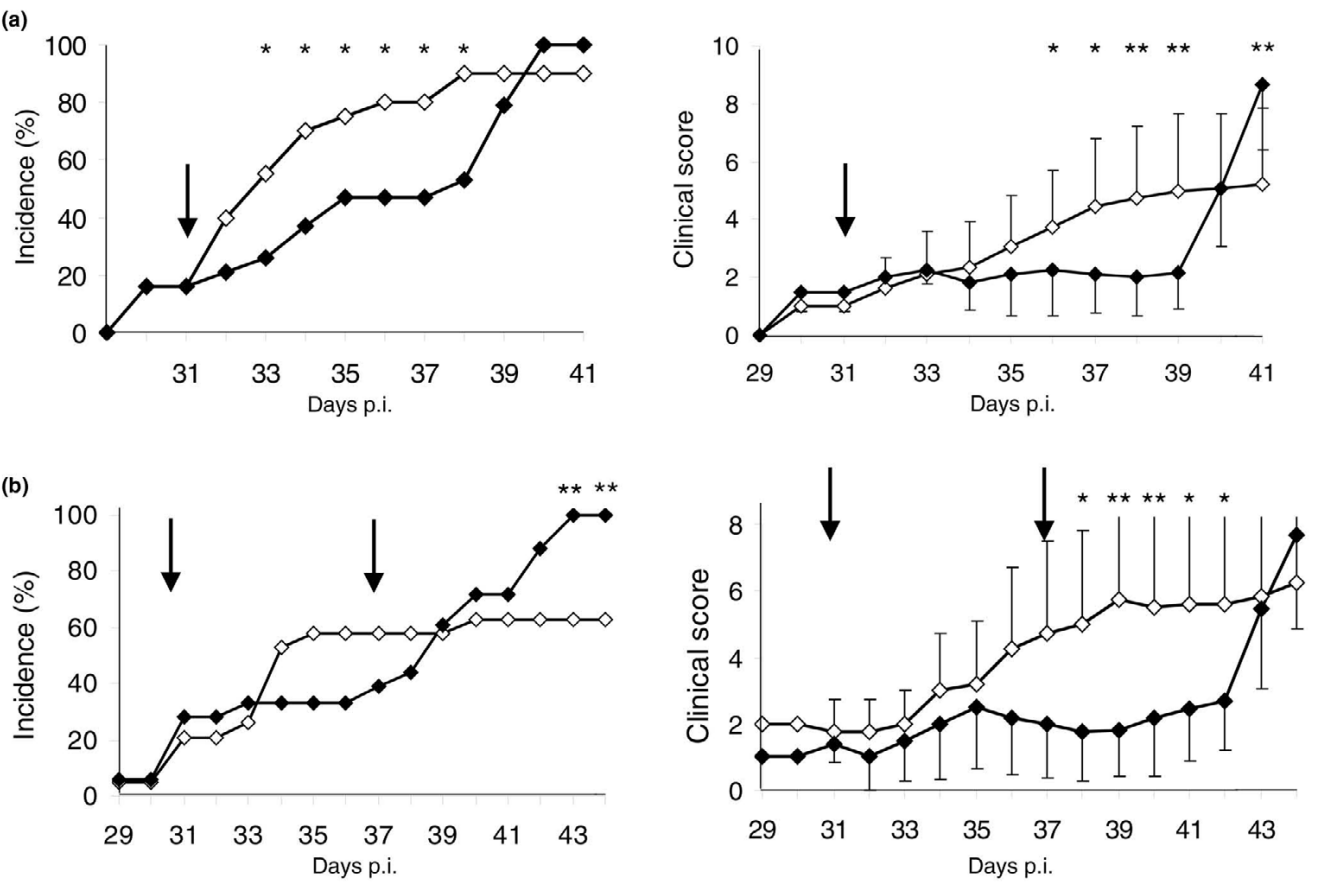
Oxaliplatin reduces both the incidence and severity of arthritis. Mice challenged with collagen type II were treated with oxaliplatin (10 mg/kg) on day 31 PI Buffered saline solutions served as controls. Significant differences were evident between the oxaliplatin treated group and the control group. Oxaliplatin *n *= 19 (▪), control *n *= 20 (▫). **(a) **An additional dose of oxaliplatin prolonged the duration of reduced arthritis severity but not the incidence of disease. **(b) **Mice challenged with collagen type II were treated with oxaliplatin (10 mg/kg) on day 31 and day 37 PI NaCl injections served as control. Significant differences in arthritis severity were evident between the oxaliplatin treated group and the control group (p < 0.05). Oxaliplatin *n *= 18 (▪), control *n *= 19 (▫). The figures demonstrate the incidence and the mean arthritis score (only sick animals included).

CIA was induced in DBA/1 mice and the animals were treated 31 days PI either IP (*n *= 19) or IV (*n *= 20) with a single dose of oxaliplatin (10 mg/kg) or vehicle alone (*n *= 19). A total of 20% of the animals had already developed clinical signs of arthritis, when therapy was initiated. Progression of disease was evaluated by accumulated incidence and clinical scores until 41 days PI (Figure 1a). A clinically beneficial, but transient, effect was mediated by oxaliplatin both regarding severity and incidence of disease. Similar effects were observed following IP administration or IV injection (data not shown). A statistically significant reduction of the incidence of arthritis was scored between day 33 and 38 and diminished clinical severity between day 36 and 39. The treatment both prevented the development of arthritis and ameliorated clinically visible disease. An abrupt clinical relapse, reminiscent of a rebound phenomenon, occurred between day 39 and 41, when oxaliplatin-exposed animals developed more severe disease than controls (p < 0.001).

A second set of experiments was conducted to determine whether repeated oxaliplatin injections would prolong the duration of clinical response. Collagen type II-challenged mice were given oxaliplatin IP (10 mg/kg) (*n *= 18) on day 31 PI, a second injection on day 37 PI and arthritis progression was followed until day 44 PI (Figure 1b). Clinical severity of inflammation was significantly reduced between day 38 and 42 in the oxaliplatin-treated group. The clinical relapse suddenly occurring between day 42 and 43 following injection in the oxaliplatin-exposed animals was as dramatic as that seen in the first experiment. No statistically verified differences between the groups were observed regarding incidence of disease during the initial 41 days following treatment. However, articular inflammation was drastically aggravated at the end of the observation period in the oxaliplatin-treated animals. All animals in the oxaliplatin-exposed group developed arthritis on day 43, when only 60% of the controls were affected (p < 0.05).

### Oxaliplatin-treated animals expressed less synovial extranuclear HMGB1 protein, less cell infiltration, and less cartilage and bone damage on day 36 PI than control-treated animals

**Figure 2:**
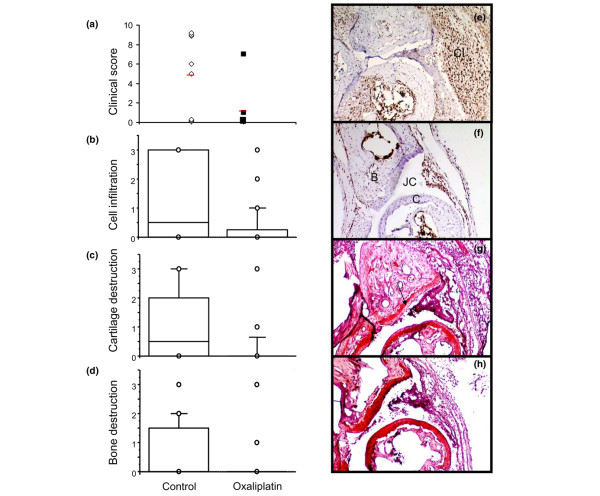
Animals treated with oxaliplatin demonstrated reduced cell infiltration, cartilage and bone destruction as well as less cytosolic and extracellular HMGB1. Oxaliplatin treated mice (*n *= 8 with a mean clinical score of 1.0) and mice given vehicle alone (*n *= 8 with a mean clinical score of 4.8) **(a) **were killed on day 36 PI and intra-articular effects of oxaliplatin were evaluated by immunohistochemistry. Cell infiltration **(b)**, cartilage **(c) **and bone **(d) **destruction were all significantly lower in oxaliplatin treated mice. Representative micrographs illustrating HMGB1 staining (in brown) in synovial tissue where an abundant cytosolic and extracellular HMGB1 staining was evident in mice treated with control vehicle **(e) **as compared to the low extranuclear HMGB1 expression in oxaliplatin treated mice **(f)**. Sequential sections stained with Safranin O demonstrating reduced proteoglycan content in articular cartilage in the control **(g) **than in oxaliplatin treated animals **(h)**. Signs of pronounced articular destructions are more evident in control treated animals, where destained cartilage layers reflected loss of matrix proteoglycans (see arrow). No cartilage destruction was detected in oxaliplatin treated mice indicated by a homogenous cartilage staining. CI, cell infiltration, JC, joint cavity, B, bone, C, cartilage. The boxes represent 25th to 75th percentiles and the lines inside the boxes stand for the median. The lines outside the boxes reflect 10th and 90th percentiles and circles indicate outliers. p = 0.05. Animals: control *n *= 8, oxaliplatin *n *= 8. Paws: control *n *= 32, oxaliplatin *n *= 32.

Sixteen mice were immunized in a separate experiment to further examine the immunohistological features of oxaliplatin treatment. The animals were treated on day 31 PI with either a single dose of oxaliplatin IP (10 mg/kg) or vehicle alone and were killed on day 36 PI. Two out of eight oxaliplatin-treated mice (mean arthritis score 1) and five of the eight control mice (mean arthritis score of 4.8) had developed clinical disease on day 36 (Figure 2a). Cellular infiltration in the joints of oxaliplatin-treated mice was significantly lower when compared to mice given vehicle alone (Figure 2b,e,f). Pro-inflammatory, extranuclear expression of HMGB1 was abundant in the synovitis of control animals (Figure 2e). It was evident that both cytoplasmic and extracellular expression of HMGB1 in the oxaliplatin-treated joints was substantially reduced compared to control animals (Figure 2e,f). In addition, articular cartilage damage (Figure 2c,g,h) and bone destruction (Figure 2d) were both significantly reduced in the oxaliplatin-treated group. In summary, these results demonstrated that reduced extranuclear HMGB1 expression coincided with beneficial oxaliplatin-induced therapy effects.

A pronounced aggravation was observed when HMGB1 staining was performed in articular tissue from four oxaliplatin treated animals killed on day 44 PI (Figure 1b). At this late timepoint, all studied animals suffered from severe clinical disease. Synovitis specimens from both oxaliplatin treated and control animals showed massive cellular and extracellular HMGB1 expression, indicating that the nuclear entrapment of HMGB1 represented a reversible process. Failure of oxaplatin-mediated protection from arthritis at this late phase of disease thus coincided with extensive extracellular HMGB1 presence.

### Oxaliplatin prevents HMGB1 but not TNF release from activated macrophage cultures

**Figure 3:**
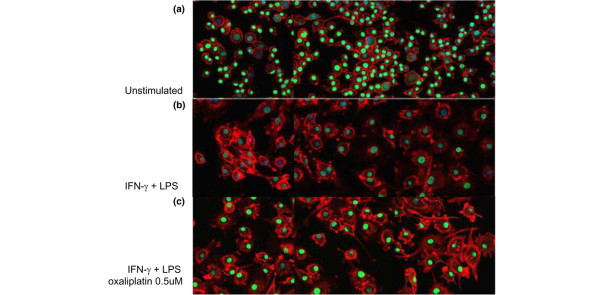
Stimulated macrophages demonstrated a nuclear retention of HMGB1 in co-cultures with oxaliplatin. RAW 264.7 macrophage-like cells were cultured without exogenous stimulus **(a) **or with IFN-γ and LPS alone **(b) **or with IFN-γ and LPS in the presence of 0.5 μM oxaliplatin **(c)**. Immunostaining for HMGB1 (green), nucleus (blue) and the cytoskeletal protein β-actin (red) was performed in fixed cells after a culture time of 24 h. Almost all nuclei demonstrated strong HMGB1 expression in resting cells **(a)**, while the nuclear HMGB1 was as expected substantially reduced in IFN-γ- and LPS-activated macrophages **(b)**. Macrophages that had been activated in the same way in the presence of oxaliplatin demonstrated a strong nuclear retention of HMGB1 **(c)**.

**Figure 4:**
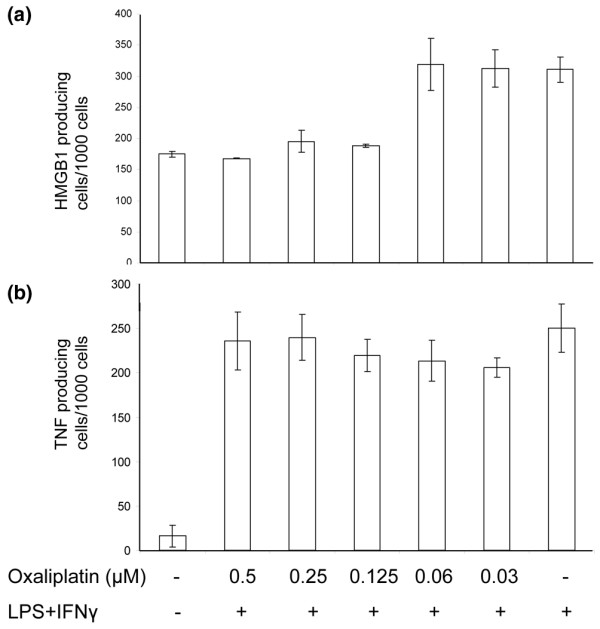
HMGB1 but not TNF release was reduced by oxaliplatin treatment. RAW 264.7 macrophage-like cells were activated with IFN-γ and LPS in HMGB1-specific or TNF-specific (24 h and 7 h respectively) Elispot plates in the absence or presence of oxaliplatin (ranging from 0.03–0.5 μM) **(a)**. Oxaliplatin inhibited HMGB1 release in a dose-dependent manner. The release of TNF was in contrast not affected **(b)**.

We performed additional experiments to further evaluate the effects mediated by oxaliplatin on the cellular localization of HMGB1 in cultured macrophages. Murine, macrophage-like RAW 264.7 cells were incubated with oxaliplatin (0.03–0.5 μM) and stimulated with LPS and IFN-γ. Co-culturing with oxaliplatin resulted in strong intranuclear retention of HMGB1 revealed by intracellular HMGB1 staining (Figure 3). Furthermore, a dose-dependent extracellular reduction of HMGB1 release in cultures containing oxaliplatin was observed using an HMGB1 Elispot assay, developed by our group (Figure 4). To determine the specificity of this inhibition, parallel experiments were performed to determine whether oxaliplatin also modulates TNF secretion in activated RAW 264.7 cells. Oxaliplatin produced divergent effects on TNF secretion as compared with HMGB1 secretion. Thus, oxaliplatin, in concentrations ranging from 0.03–0.5 μM did not affect LPS+IFN-γ-induced TNF secretion from RAW 264.7 cells (Figure 4).

### Oxaliplatin inhibits antigen-induced proliferation in cultured lymph node T lymphocytes from DBA/1 mice

**Figure 5:**
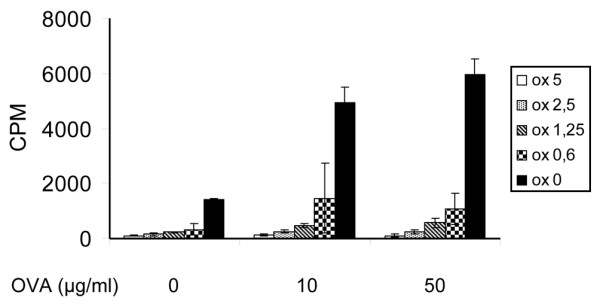
T-cell proliferation was abrogated by oxaliplatin treatment. T-cell proliferation assay was performed with lymph node cells from DBA/1 mice immunized with ovalbumin (OVA) Oxaliplatin was added to the cell culture and the proliferation was measured. Oxaliplatin suppressed the proliferative response in a dose-dependent manner when compared with controls.

Since induction of CIA is a T lymphocyte-dependent process we also performed studies of the influence of oxaliplatin on T-cell activation in the animal model. Ovalbumin was used to challenge the proliferative response in LN cells. We used ovalbumin since CII is known to be a poor inducer of T-cell proliferation. Cultures with lymph node cells were strongly suppressed by oxaliplatin in doses tested ranging from 0.6 to 5 μM (Figure 5). We did not study whether oxaliplatin–HMGB1 interactions played any functional role in the observed anti-proliferative effects.

## Discussion

HMGB1 is an endogenous pathogenic factor in synovitis, and the present results that oxaliplatin-induced nuclear HMGB1 sequestration coincided with beneficial therapy outcome support this notion. Several research groups including ours have previously presented analogous conclusions based on results obtained with extracellular HMGB1 antagonists. *In vitro *use of oxaliplatin levels relevant to plasma concentrations obtained with therapy of patients with neoplastic disease, demonstrated in the present study, show that the extracellular release of HMGB1 from activated macrophage-like cells was substantially suppressed. Immunostaining and Elispot assessment indicated that nuclear retention of HMGB1 coincided with inhibited extracellular HMGB1 release. In addition, we have recent data from oxaliplatin-independent experiments supporting the concept that nuclear HMGB1 retention may counteract inflammation [34]. Activated macrophages that were co-cultured with gold salts expressed pronounced nuclear HMGB1 sequestration and reduced cytoplasmic and extracellular HMGB1 release. Gold compounds may induce sustained remission of synovitis in subgroups of patients with rheumatoid arthritis, but the mechanisms are poorly understood. It is a distinct possibility that prevention of extracellular HMGB1 release forms a common denominator for the action of gold and the therapeutic effects exerted by oxaliplatin in the present studies.

In contrast to these findings, parallel *in vitro *studies demonstrated that oxaliplatin did not block TNF release. The fact that TNF production was not blocked by oxaliplatin argue against oxaliplatin-mediated cell toxicity or cell death as the basis for decreased HMGB1 secretion. In the absence of an effect of oxaliplatin on cell viability or apotosis, oxaliplatin inhibition of extracellular HMGB1 release appears to reflect disruption in the intracellular processing and trafficking of this protein leading to its nuclear retention.

Clinically beneficial results were obtained with oxaliplatin treatment as long as HMGB1 was mainly retained intranuclearly and unable to reach the extracellular compartment. During later stages of the observation period, when clinical relapse occurred, an extensive cytoplasmic and extracellular localization of HMGB1 was obvious. We can only speculate about the mechanisms responsible for the dramatic rebound effects occurring in both experiments in the oxaliplatin-treated groups. We suggest that the nuclear HMGB1 entrapment in oxaliplatin-treated animals constituted a reversible process, and that massive HMGB1 release may have occurred within a short period of time later, especially if cells underwent non-apoptotic cell death. An alternative explanation could be that the origin of the pro-inflammatory extracellular HMGB1 came from novel, oxaliplatin-unexposed cells that were recruited to the articular compartment. Histological examination of synovial tissue did not demonstrate massive apoptotic or necrotic cell death in oxaliplatin-treated animals when compared to controls as a possible explanation (data not shown).

The focus of this therapeutic intervention study is on HMGB1 biology, but it is equally feasible that the beneficial therapeutic effects mediated by oxaliplatin on synovitis can be partly explained by additional cellular mechanisms. For example, our *in vitro *studies indicated that antigen-driven T-lymphocyte proliferation was strongly inhibited by oxaliplatin. This is a significant finding, since induction of collagen type II-induced synovitis requires T cell help. It is presently unknown to what extent the impaired proliferative capacity of T cells is an HMGB1 dependent process. In any case, inhibiting extracellular HMGB1 release to combat synovitis remains a promising strategy. Future work on agents that can selectively regulate intracellular HMGB1 transport mechanisms will be needed to study this as a novel therapeutic possibility.

## Conclusion

Our study demonstrates a novel strategy of treatment of arthritis by nuclear sequestration of HMGB1 thereby preventing HMGB1 release. This finding may reveal an important functional role of HMGB1 in the pathogenesis of arthritis. The results also support a search for additional compounds preventing HMGB1 release selectively to treat inflammatory diseases.

## Abbreviations

CIA = collagen-induced arthritis; HMGB1 = high mobility group box chromosomal protein 1; IFN = interferon; IL = interleukin; LN = lymph node; LPS = lipopolysaccharide; OVA = ovalbumin; RAGE = receptor for advanced glycation end products; TNF = tumor necrosis factor.

## Competing interests

The authors declare that they have no competing interests.

## Authors' contributions

TÖ carried out the *in vivo *studies, performed the T-cell proliferation, and the manuscript preparation. HW carried out the Elispot assays. KP carried out and analyzed the immunohistochemical studies. NI performed the immunocytochemistry assays. PS performed the statistical analysis. MS participated in the design of the study. MTL participated in the design of the study. HEH participated in the design of the study and manuscript preparation. UA participated in the design and coordination of the study and manuscript preparation. All authors read and approved the final manuscript.
